# Optimizing Object Detection Algorithms for Congenital Heart Diseases in Echocardiography: Exploring Bounding Box Sizes and Data Augmentation Techniques

**DOI:** 10.31083/j.rcm2509335

**Published:** 2024-09-19

**Authors:** Shih-Hsin Chen, Ken-Pen Weng, Kai-Sheng Hsieh, Yi-Hui Chen, Jo-Hsin Shih, Wen-Ru Li, Ru-Yi Zhang, Yun-Chiao Chen, Wan-Ru Tsai, Ting-Yi Kao

**Affiliations:** ^1^Department of Computer Science and Information Engineering, Tamkang University, 251301 New Taipei, Taiwan; ^2^Congenital Structural Heart Disease Center, Department of Pediatrics, Kaohsiung Veterans General Hospital, 813414 Kaohsiung, Taiwan; ^3^Structural/Congenital Heart Disease and Ultrasound Center, Children’s Hospital, China Medical University, 404 Taichung, Taiwan; ^4^Department of Information Management, Chang Gung University, 333 Taoyuan, Taiwan; ^5^Kawasaki Disease Center, Kaohsiung Chang Gung Memorial Hospital, 83301 Kaohsiung, Taiwan

**Keywords:** congenital heart disease, echocardiography, deep learning, object detection, data augmentation

## Abstract

**Background:**

Congenital heart diseases (CHDs), particularly atrial and ventricular septal defects, pose significant health risks and common challenges in detection via echocardiography. Doctors often employ the cardiac structural information during the diagnostic process. However, prior CHD research has not determined the influence of including cardiac structural information during the labeling process and the application of data augmentation techniques.

**Methods:**

This study utilizes advanced artificial intelligence (AI)-driven object detection frameworks, specifically You Look Only Once (YOLO)v5, YOLOv7, and YOLOv9, to assess the impact of including cardiac structural information and data augmentation techniques on the identification of septal defects in echocardiographic images.

**Results:**

The experimental results reveal that different labeling strategies substantially affect the performance of the detection models. Notably, adjustments in bounding box dimensions and the inclusion of cardiac structural details in the annotations are key factors influencing the accuracy of the model. The application of deep learning techniques in echocardiography enhances the precision of detecting septal heart defects.

**Conclusions:**

This study confirms that careful annotation of imaging data is crucial for optimizing the performance of object detection algorithms in medical imaging. These findings suggest potential pathways for refining AI applications in diagnostic cardiology studies.

## 1. Introduction 

Echocardiography is often used in clinical practice to determine cardiac function. Echocardiographic findings can assist cardiologists in performing additional diagnostic tests and selecting appropriate treatments. However, considerable clinical experience is required for doctors to identify cardiac diseases and make sound decisions on the basis of imaging findings to identify cardiac diseases. Therefore, the present study identified a method that assists in the identification of cardiac diseases, particularly congenital heart diseases (CHDs), by echocardiographic images. One of the most commonly encountered types of congenital heart defects is a ventricular septal defect (VSD) [1]. Wu *et al*. [2] reported that VSDs and atrial septal defects (ASD; secundum) are the two most common CHDs in Taiwan. Veronese *et al*. [3] demonstrates the accuracy of the Fetal Intelligent Navigation Echocardiography (FINE) method in detecting atrioventricular septal defects in pregnancies, supporting early diagnosis and treatment decisions.

**Fig. 1.**
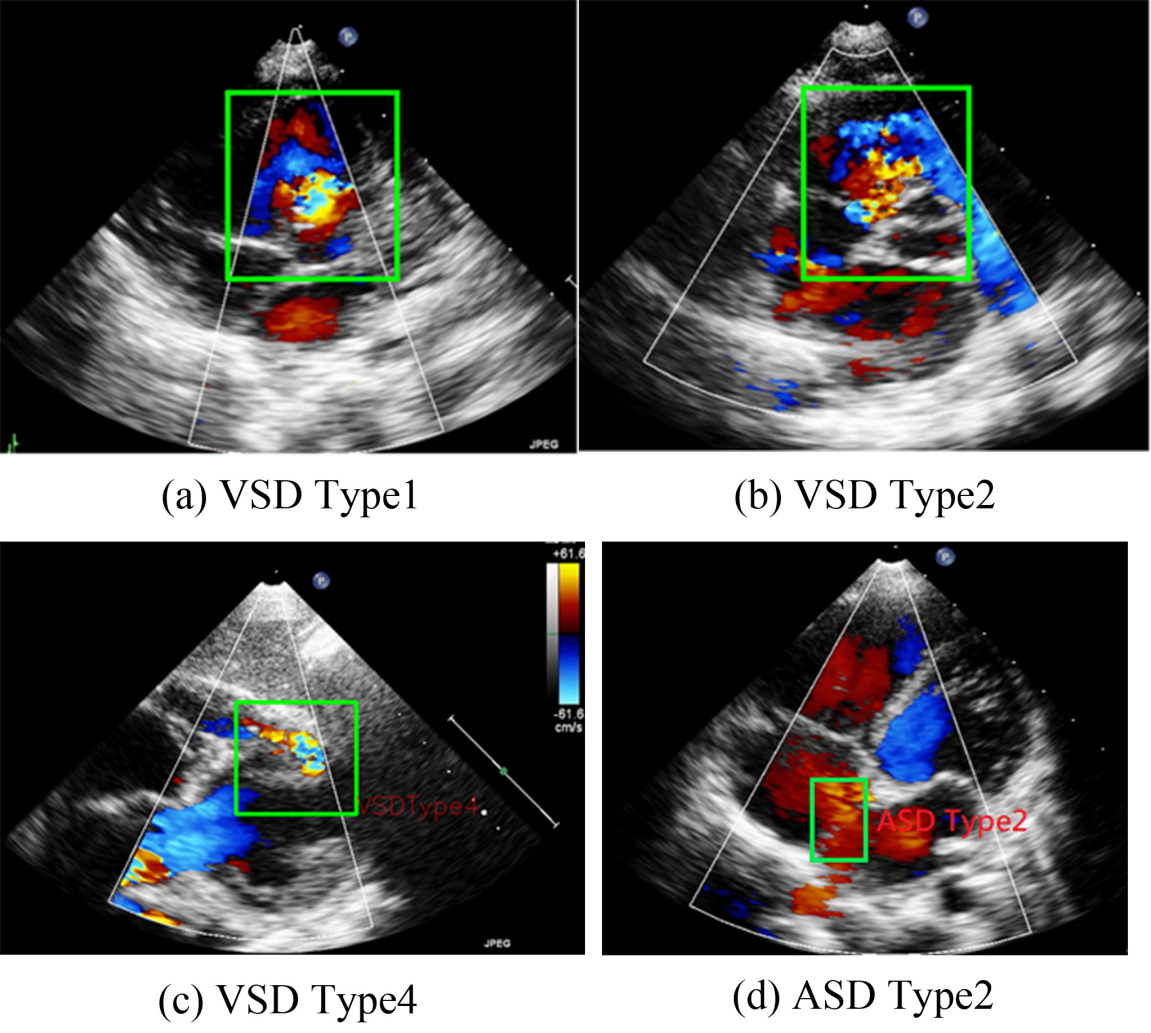
**Representations of various ventricular septal defect (VSD) types and atrial septal defects (ASD) Type 2**. (a–c) are the three types of VSD. (d) shows a typical pattern of ASD Type 2.

VSD can be categorized into four types on the basis of its location in the right ventricular septum [4]. The present study focuses on Type II VSDs. Type II VSD is a membranous VSD [Fig. 1b] located in the interventricular area of the membranous septum. Membranous VSD is the most common type of VSD, accounting for more than 50% of VSD cases. The recommended interventions for VSD are those outlined in the guidelines proposed by Marelli *et al*. [1].

ASD occurs in the atrial septum. As illustrated in Fig. 1, ASD secundum (or Type II ASD) is the most common ASD type and accounts for approximately 80% of ASD cases. Type II ASD is generally caused by the enlargement of the foramen ovale and the inadequate development of the septum secundum.

**Fig. 2.**
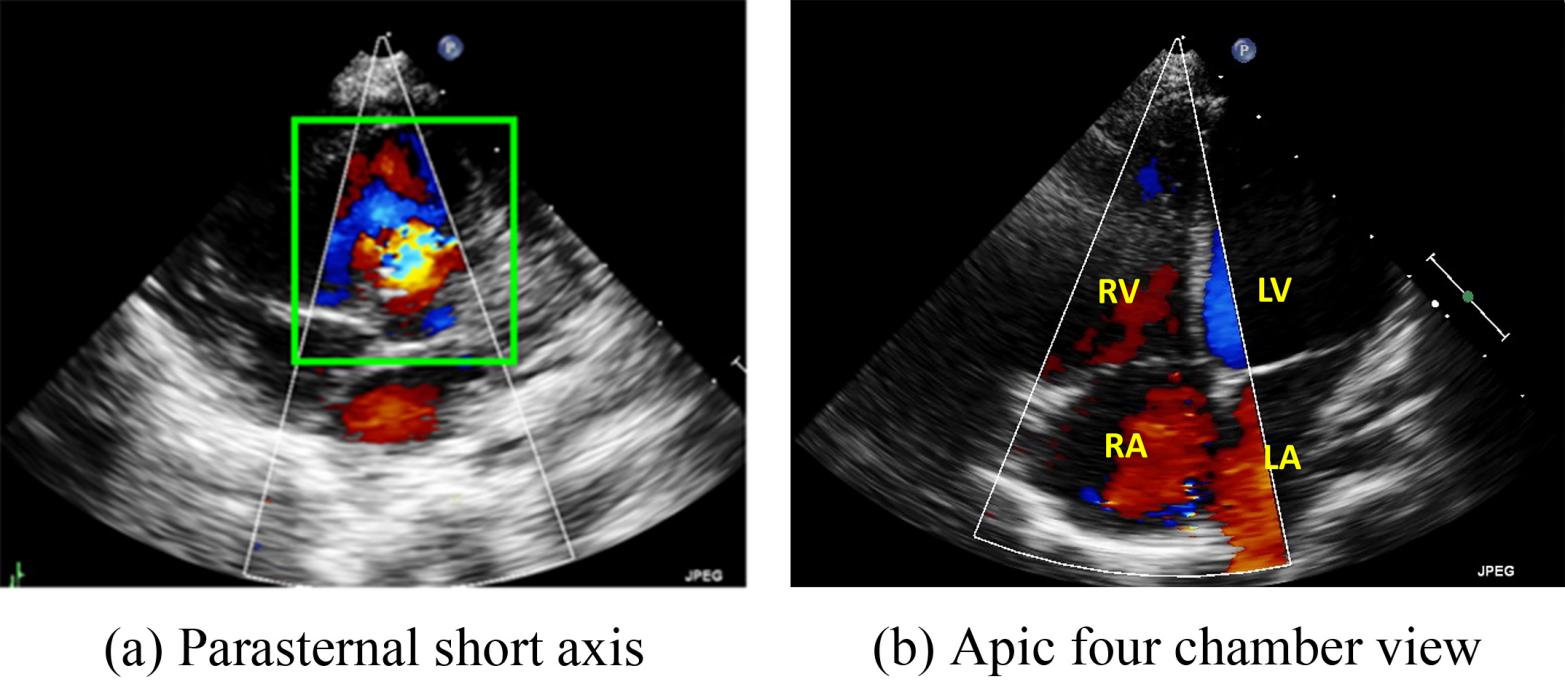
**Two major echo views often used by doctors to diagnose the ventricular septal defect (VSD) and atrial septal defects (ASD)**. (a) Parasternal short-axis view. (b) Four chamber view. RV, right ventricle; LV, left ventricle; RA, right atrium; LA, left atrium.

In this study, we focused on Type II ASDs because it is the most common type. For our analysis, we used ultrasound images in the parasternal left ventricular short-axis view: the aortic root section, four apical AV sections, five apical AV sections, the short-axis section of the two cavities under the xiphoid process, and the long-axis section of the two cavities under the xiphoid process (Fig. 2).

Deep-learning has been widely used for medical image classification, object-detection, and segmentation [5, 6, 7]. Deep-learning has been applied in pediatric echocardiography, for the detection of CHDs [4, 8, 9, 10]. However, further studies exploring the potential applications of deep-learning in the field of object-detection are required [4]. Although object-detection algorithms can be used to determine edge information, defining the edge of a clinically anomalous object on ultrasound images remains challenging. In this study, to determine the bounding box size for the recognition of ASDs and VSDs in medical images, we defined two sizes: one where the box is a large one that includes the structures neighboring a lesion (i.e., the nearby atrium or ventricle), and one where the box is a small one that excludes such a structure.

Several augmentation methods can be used to increase the amount of data available to train machine-learning and deep-learning algorithms. The most common methods include flip, rotation, scale, brightness, contrast, cropping, and cutout [11]. In flip augmentation, new images are artificially created by flipping the original images horizontally or vertically. However, vertically or horizontally flipping echocardiographic images changes the location of the chamber, which reduces the likelihood of over-fitting but at the cost of lower prediction performance. The present study conducted experiments to investigate the effects of employing left-right flip augmentation for echocardiographic images.

We evaluated the influence of the structures around a lesion and the use of flip augmentation on the performance of two well-known object-detection algorithms: You Look Only Once (YOLO)v5 [12], YOLOv7 [13], and YOLOv9 [14]. Both algorithms were trained using the same data sets with and without the inclusion of the structures neighboring a lesion and when the augmentation method was employed. The following section describes the methods used in this study.

## 2. Research Methods

The algorithms used in this study, namely the YOLOv5 and YOLOv7, are based on YOLOv4, which is described in Section 2.1. In addition, we present two potential methods for determining bounding boxes and augmentation in Section 2.2.

### 2.1 Main Characteristics of YOLOv5, YOLOv7 and YOLOv9

Arising from YOLOv4 [11], Jocher *et al*. [12] proposed YOLOv5 which continues the YOLO tradition, balancing performance and efficiency. It employs a Cross Stage Partial Network (CSPNet) backbone, enhancing learning while reducing complexity, and integrates a Path Aggregation Network (PANet) neck for effective multi-scale feature fusion. The model’s head uses anchor-based detection with optimized anchor box clustering for precise object localization and classification. YOLOv5’s training combines Cross-Entropy, Generalized Intersection over Union (GIoU), and Objectness Losses, supplemented by data augmentation and adaptive learning rate strategies to improve robustness and generalization. YOLOv5 has five model sizes ranging from small to large: YOLOv5s, YOLOv5m, YOLOv5l, and YOLOv5x. YOLOv5s is the smallest model and thus has the highest speed but the lowest accuracy. Among these models, the accuracy increases and the speed decreases with the size of the model.

YOLOv7 [13] introduces innovations such as the trainable bag-of-freebies, extended Extended Efficient Layer Aggregation Network (ELAN), and RepVGG architecture to enhance detection without increasing computational load, to increase efficiency. These advancements, including model re-parameterization and dynamic label assignment, significantly reduce the model’s parameters and computation, maintaining high accuracy, making YOLOv7 exceptionally competitive across detection tasks.

Compared with YOLOv7, YOLOv9 has the Programmable Gradient Information (PGI) and Generalized Efficient Layer Aggregation Network (GELAN). PGI effectively preserves more information during the training process, enhancing its ability to detect objects. GELAN is a new lightweight network architecture designed to enhance information integration and transmission efficiency in deep learning models. Its core concept is to optimize message transmission with an effective hierarchical aggregation mechanism, boosting model performance and efficiency.

Because both YOLOv5, YOLOv7, and YOLOv9 are well-known, contemporary object-detection algorithms. We compared the effects of the scale of the bounding box methods and augmentation on the object-detection precision when each algorithm was employed.

### 2.2 Bounding Box Scale and Augmentation Methods

**Fig. 3.**
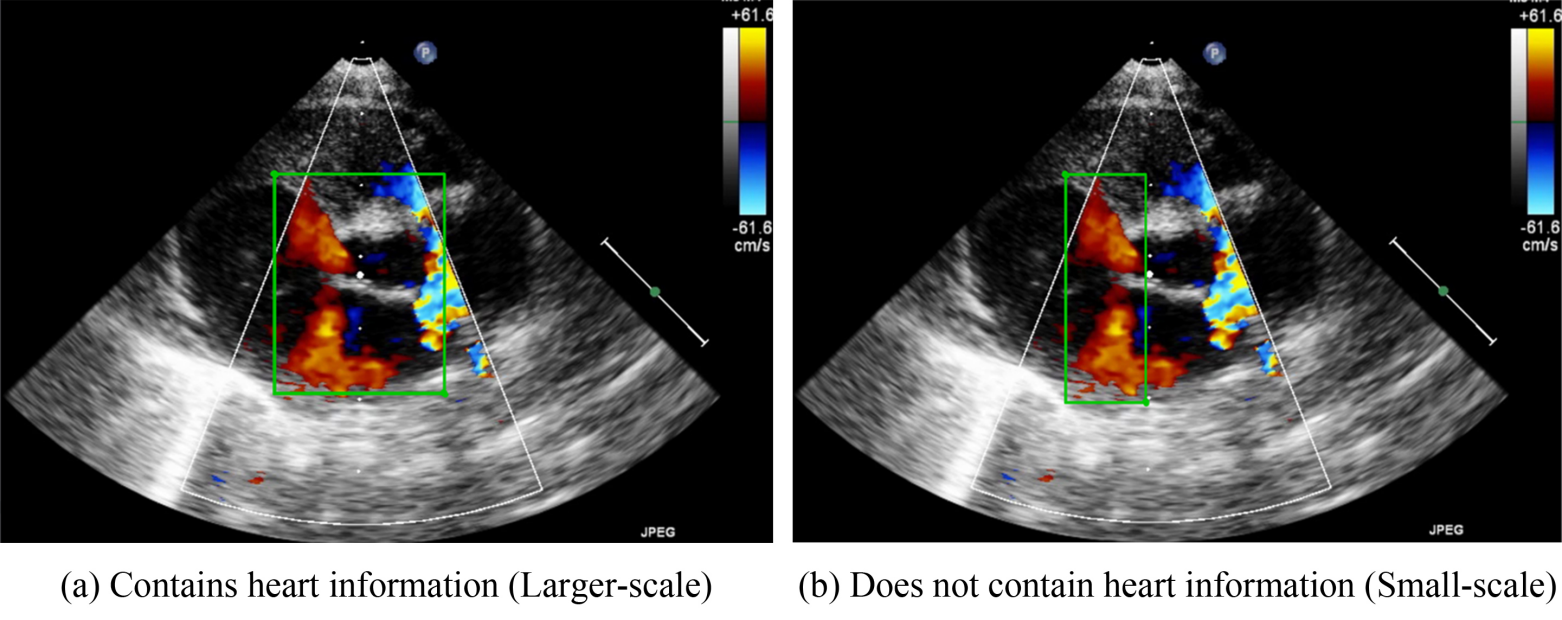
**Schematic diagram of the marking ranges**. (a) includes the heart structure of ventricular and atrium diaphragm. (b) only focuses on the jet area.

Because VSDs and ASDs require clear edges for detection, no standards for how these defects should be annotated have been developed. Therefore, the present study developed two methods for determining bounding box sizes for VSDs and ASDs. The first involves including the heart structure in echocardiographic figures. Fig. 3a, for example, presents the parasternal short-axis view of a heart. The bounding box includes the aorta in the center of the image. If an image is captured in the apical four chamber view, the bounding box should include the chamber septums. This standard was developed to ensure that the deep-learning algorithms would be able to consider the structure of the heart. Because the bounding box incorporates the heart structure, the size of the bounding box is larger. Therefore, in our study, we refer to this method as the large-scale bounding box method.

The other annotation method involves the color Doppler jet area. Fig. 3b depicts the narrow area in which diseases may be detected. Applying this method would enable doctors to focus on a specific region. Because the size of the bounding box is smaller when this method is employed, we refer to this method as the small-scale bounding box method.

In addition to the size of the bounding box, data augmentation might influence the performance of an algorithm. Data augmentation is a technique used to artificially increase the number of figures in a data set to improve the robustness and generalizability of a model. In the present study, we employed left-right flip augmentation with a probability of 0.5. We compared whether the differences in the images would affect the training results of the model under the same conditions compared with other conditions.

## 3. Experimental Findings

This research leverages deep learning techniques, specifically YOLOv5, YOLOv7, and YOLOv9 to identify VSDs and ASDs in echocardiographic images. The study delves into how the presence or absence of structures adjacent to lesions influences the accuracy of image annotations and the effectiveness of augmentation strategies. We demonstrate how to organize the experiments in Section 3.1 and the results are shown in Section 3.2.

### 3.1 Study Design

Echocardiograms in mp4 format were converted into static images and anonymized to protect patient privacy. The images were categorized based on patient conditions, verified by a physician for accuracy, and labeled using LabelMe. The dataset comprised 491 Doppler ultrasound images of ASDs and 345 of VSDs, divided into 70% training, 20% validation, and 10% testing subsets.

The training of the select three models was conducted on the Taiwan Computing Cloud (TWCC), harnessing the computational power of a Tesla V100 GPU with 32 GB of memory. The models were trained using images with a resolution of 448 × 448 pixels, over 300 epochs, and a batch size of 32, within an environment powered by the NVIDIA pytorch-22.08-py3 container image, specifically optimized for deep learning applications.

### 3.2 Results

To determine the optimal settings for detecting ASDs and VSDs, each experiment was performed three times, with the mean values serving as the final training results that would be used in subsequent comparisons (Table 1). Twelve experimental combinations involving three factors (i.e., the object-detection method, labeling scale, and left–right flip augmentation) were used in this study. We highlight the best result of each algorithm in bold. With regard to the object-detection method, the mean average precision at a 0.5 intersection over the union (mAP@.5) was used. YOLOv5 and YOLOv9 shows strong performance in large-scale settings, slightly decreasing in smaller-scales. The left–right flip improves its small-scale performance. YOLOv7 excels in large-scale performance with flip augmentation, reaching the highest mAP@.5, but has a notable drop in small-scale labeling without flip. As a result, both algorithms benefit from the large labeling scale. In terms of the flip parameter, especially in small-scale scenarios, YOLOv7 resulted in a significant improvement in large-scale detection accuracy.

**Table 1.** **Descriptive statistics of three replicate studies**.

Algorithm	Left–right flip parameter	Labeling scale	mAP@.5
YOLOv5	0.0	Large-scale	98.7%
YOLOv5	0.0	Small-scale	92.9%
YOLOv5	0.5	Large-scale	98%
YOLOv5	0.5	Small-scale	94.5%
YOLOv7	0.0	Large-scale	98.67%
YOLOv7	0.0	Small-scale	76.4%
YOLOv7	0.5	Large-scale	99.2%
YOLOv7	0.5	Small-scale	93.5%
YOLOv9	0.0	Large-scale	98.7%
YOLOv9	0.0	Small-scale	94.1%
YOLOv9	0.5	Large-scale	99.4%
YOLOv9	0.5	Small-scale	94%

YOLO, You Look Only Once.

To ascertain the most effective configurations for ASD and VSD detection, each experiment was replicated three times, and the average of these outcomes was employed for subsequent analyses, as detailed in Table 1. The study explored eight different combinations of three variables: the object-detection algorithm used, the scale of labeling, and the implementation of left–right flip augmentation. To assess object detection performance, the mean average precision at an intersection over a union (mAP@.5) threshold of 0.5 was utilized. YOLOv5, YOLOv7 and YOLOv9 showed robust performance in large-scale labeled settings, though its effectiveness marginally decreased in smaller scales. The introduction of left–right flip augmentation significantly enhanced the performance of YOLOv5 in small-scale environments. Conversely, YOLOv7 demonstrated superior performance in large-scale scenarios with flip augmentation, achieving the highest mAP@.5, but experienced a significant reduction in accuracy for small-scale labels without augmentation. These results underscore the importance of labeling scale and augmentation techniques, particularly flip augmentation, in optimizing detection accuracy for both YOLOv5, YOLOv7 and YOLOv9 models.

Statistical analysis, conducted using Matlab software (version 23.2, MathWorks, Natick, MA, USA), assessed the effects and interactions of Method, Area, and Augmentation on detection performance, as detailed in Table 2. The “Method” factor denotes the utilized algorithm, “Area” refers to whether an area including the heart was marked, and “Augmentation” signifies the application of left-right image flip. The analysis of variance (ANOVA) results demonstrate significant impacts of these factors on outcomes, with *p*-values all below 0.001, providing robust evidence against the null hypothesis. Furthermore, significant interactions among Method, Area, and Augmentation indicate that these variables intricately influence detection accuracy. The effect size, measured by the F-value—a statistic indicating the ratio of variance between groups to the variance within groups—was largest for Area, followed by the Augmentation, and the combination effects of Method and Area, emphasizing the need to consider these elements and their interplay in the analysis.

**Table 2.** **ANOVA table of the main effect factors and interactions among the factors**.

Source	Sum Sq.	d.f.	Mean Sq.	F	Prob > F
Method	0.01527	2	0.00764	14.91	0
Area	0.05585	1	0.05585	109.03	0
Augmentation	0.00915	1	0.00915	17.87	0.0003
Method*Area	0.01679	2	0.00840	16.39	0
Method*Augmentation	0.01426	2	0.00713	13.91	0.0001
Area*Augmentation	0.00816	1	0.00816	15.93	0.0005
Error	0.01332	26	0.00051		
Total	0.13281	35			

ANOVA, analysis of variance.

**Fig. 4.**
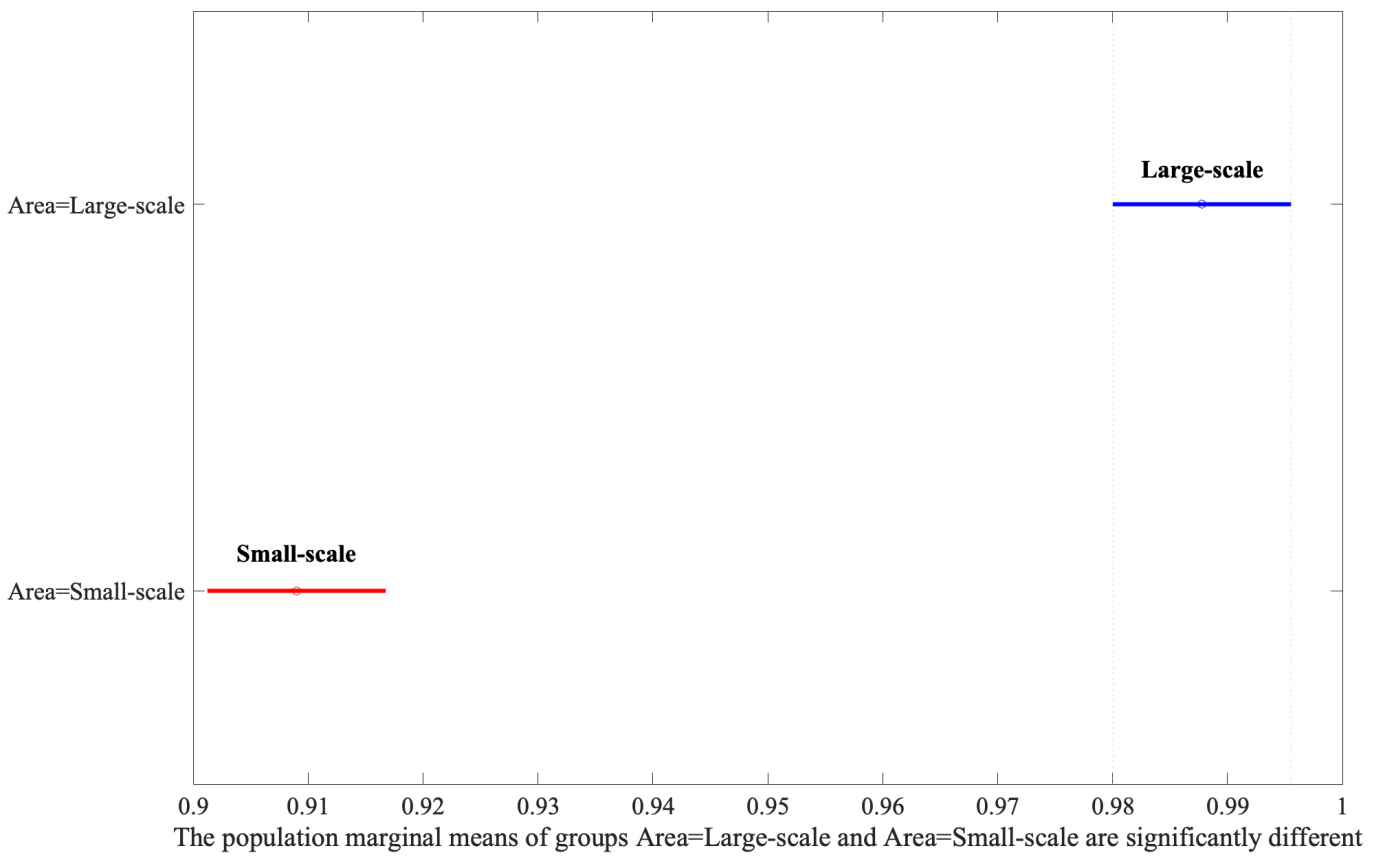
**Analysis of area variations using Least Significant Difference (LSD) post-hoc test**.

**Fig. 5.**
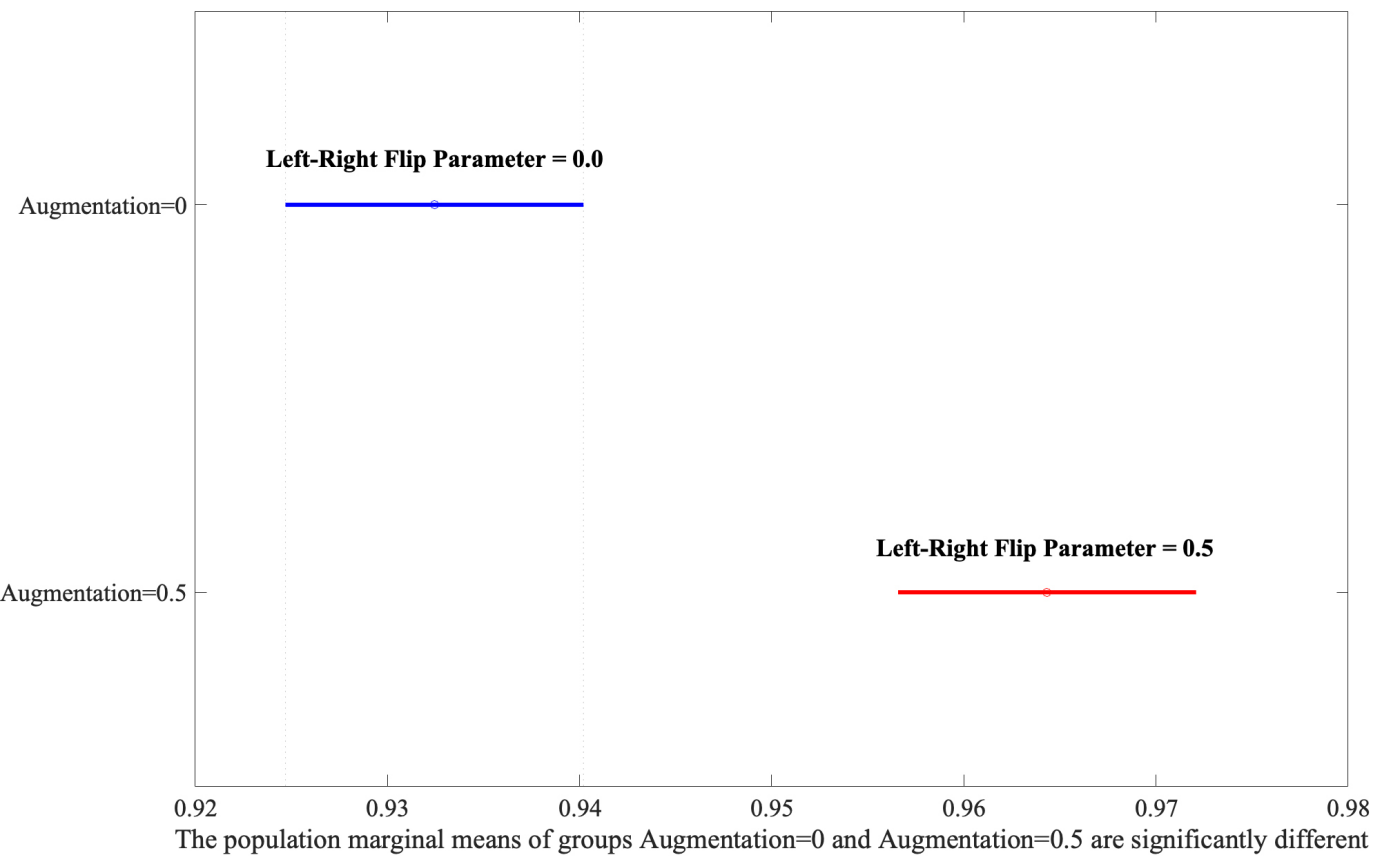
**Exploring the interaction between Area and Augmentation through post-hoc analysis**.

Following the identification of significant factors in the ANOVA analysis, the Honestly Significant Difference (HSD) test was employed to determine specific group mean differences. This post-hoc analysis, vital for pinpointing precise contrasts between factor levels, revealed that Large-Scale bounding areas significantly outperform Small-Scale ones, as shown in Fig. 4. Moreover, the Augmentation yielded the second highest F-value, illustrating that the application of the flip parameter set to 0.5 has better performance depicted in Fig. 5.

**Fig. 6.**
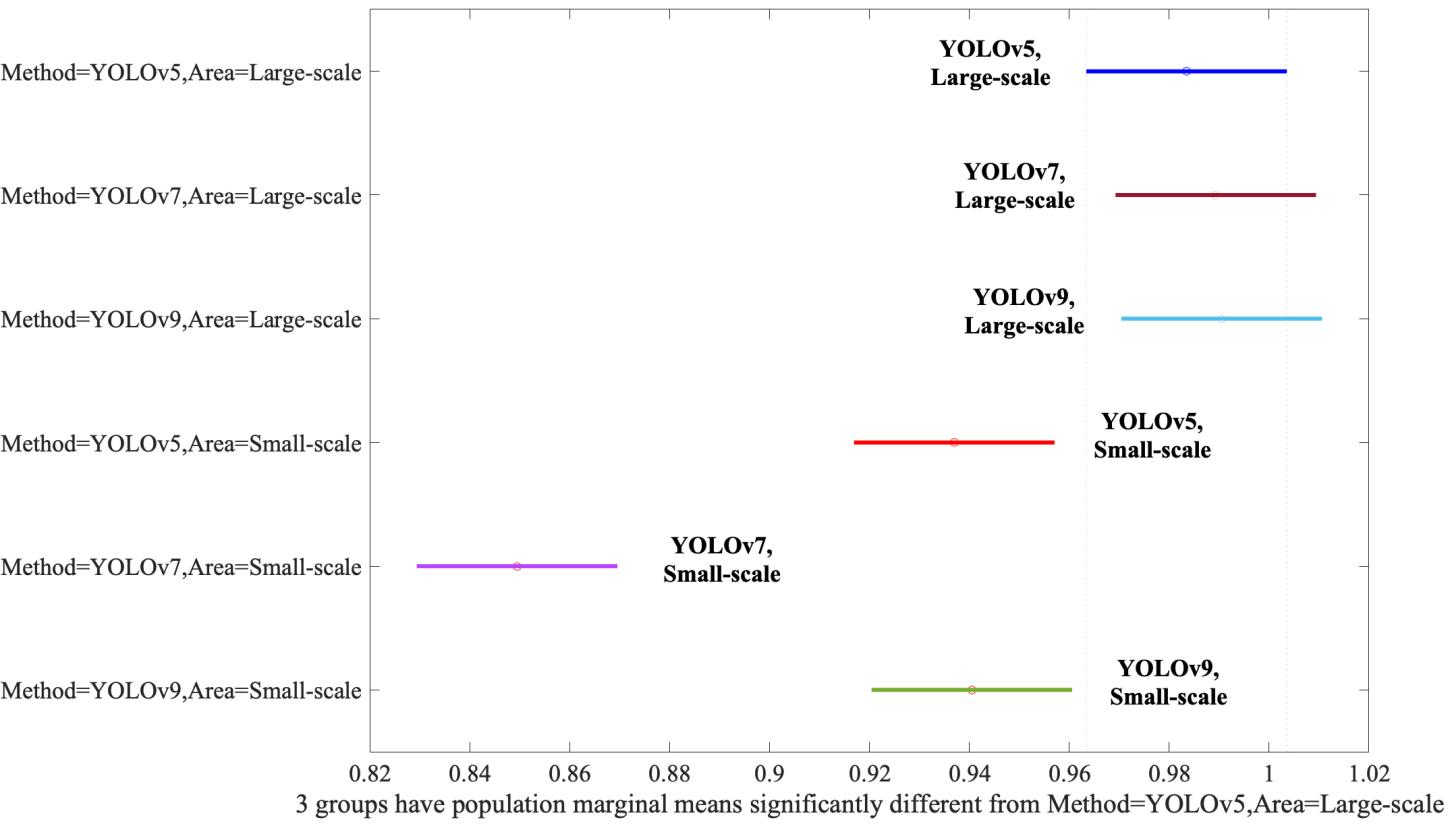
**Assessment of the Method and Area interaction via post-hoc analysis**. YOLO, You Look Only Once.

Finally, we need to evaluate the interaction between the Method and Area. When YOLOv5, YOLOv7, and YOLOv9 models were applied to Large-Scale bounding boxes, no discernible difference in efficacy was observed between the algorithms, as evidenced by Fig. 6, underscoring their comparable performance under these conditions. YOLOv5, YOLOv7, and YOLOv9 performed statistically equally even though the YOLOv9 was slightly better than the YOLOv5 and YOLOv7. However, if the algorithms applied the Small-Scale bounding boxes, the performances were degraded significantly.

## 4. Discussion

Numerous CHD studies have investigated the application of artificial intelligence for pediatric echocardiography [4, 8, 9, 10, 15, 16]. For example, a study [10] proposed a multiview classification model for diagnosing ASDs and VSDs Nurmaini *et al*. [9] proposed a deep-learning-based, computer-aided method for fetal-heart echocardiographic image analysis with an instance segmentation approach to improve the accuracy of detecting structural abnormalities, such as congenital heart defects. The results indicated that the method had satisfactory performance in segmenting images in the standard views and in detecting congenital heart defects. Arnaout *et al*. [8] investigated an ensemble of neural networks by training an algorithm with 107,823 images created from 1326 fetal echocardiograms to identify the optimal cardiac views for distinguishing normal hearts from those with complex CHDs, such as the tetralogy of Fallot and hypoplastic left heart syndrome.

Chen *et al*. [4] proposed YOLOv4-DenseNet to solve the object-detection problem for detecting three VSD subtypes. Xu *et al*. [16] proposed a deep-learning framework that combines deep-learning and graph algorithms for whole-heart and great-vessel segmentation in images of hearts with CHDs to overcome the ineffectiveness of whole-heart and great-vessel segmentation frameworks when they are applied for medical images in hearts with significant variations in heart structure and great-vessel connections. Liu *et al*. [15] explored a new deep-learning algorithm model for screening and diagnosing specific types of left-to-right shunt CHDs, such as ASD, VSD, and patent ductus arteriosus, by using electrocardiographic data. Truong *et al*. [17] extracted cardiac information and investigated whether the random forest algorithm would improve the sensitivity of predicting the presence or absence of CHDs in fetal echocardiographic images and demonstrated that six essential features play crucial roles in the prediction of such diseases. However, none of these studies considered the influence of the structures neighboring a lesion.

In the above studies, the structures neighboring a lesion are the surrounding tissues or organs that are adjacent to a lesion, and were not considered. The results of the present study’s post-hoc analysis (Fig. 4) indicated that including the structures neighboring a lesion in image labeling improved the performance of both the YOLOv5 and YOLOv7 object-detection algorithms. These results indicate that such neighboring structures provide key information about a lesion, such as its location and relationship to other structures, and that the inclusion of this cardiac information is key when labeling bounding boxes.

Overall, our findings reveal that the structures neighboring a lesion should be included for both object-detection algorithms when training data sets are being labeled. In addition, the left-right flip augmentation is no longer sensitive during the training phase. Once we select the structures neighboring a lesion in image labeling, the average mAP value of the object detection algorithm was higher than 98% no matter which left-right flip was used. As a result, the performance can be considered satisfactory. Because doctors might consider memorizing and recalling the large volumes of information required for object-detection to be difficult [18], designing an effective object-detection algorithm could be of considerable assistance.

There are three limitations of this paper. Firstly, this paper presents the importance of inclusion of structures neighboring a lesion for other medical image recognition problems. Even though this approach might cause some difficulties in labeling the dataset, the labeling process may adopt the expertise from experienced doctors. Secondly, our experimental results show the inclusion of structures neighboring a lesion is effective for the two selected algorithms, future research needs to evaluate this concept of the proposed algorithm. Finally, the potential challenges in actual clinical environments may include the insufficient computing ability and that the software environment is hard to establish in existing ultrasound machines. Hence, it might require ultrasound machine manufacturers to re-design the equipment.

## 5. Conclusions

The present study highlights the importance of employing appropriate image-labeling and data augmentation techniques for achieving accurate results in detecting VSD and ASD in echocardiographic images. We applied two well-known object-detection algorithms, YOLOv5, YOLOv7 and YOLOv9, to validate our results. Overall, labeling including the structures neighboring lesions led to more favorable training outcomes than small-scale labeling. Inclusion of information on nearby areas more effectively improved image recognition than when this information was excluded. When the training data for the object-detection algorithms included structures neighboring lesions, both well-known algorithms achieved a higher mean average precision score compared with the labeling without including the heart structure information. In future research, neighboring structure inclusion and flip augmentation can be applied for the training data sets for more recently developed object-detection algorithms, such as YOLOv10 [19], to improve their performance.

## Availability of Data and Materials

The data is available upon request. Please send an email to shchen@mail.tku.edu.tw to request access.
